# Is leaf area of Norway spruce (*Picea abies* L. Karst.) and European larch (*Larix decidua* Mill.) affected by mixture proportion and stand density?

**DOI:** 10.1007/s13595-016-0614-x

**Published:** 2017-02-06

**Authors:** Gerald Dirnberger, Angela-Elisabeth Kumer, Eduard Schnur, Hubert Sterba

**Affiliations:** Department of Forest and Soil Sciences, Institute of Forest Growth, BOKU University of Natural Resources and Life Sciences, Peter-Jordan-Straße 82, 1190 Vienna, Austria

**Keywords:** Leaf biomass, Crown allometry, Crown surface area, Mixed stands

## Abstract

*****Key message***:**

**Trees with otherwise equal dimensions have different leaf areas if they are located in different stand types. While leaf area of European larch is affected by mixture proportion, leaf area of Norway spruce is affected by stand density.**

*****Context***:**

Leaf area is a key parameter for evaluating growth efficiency of trees, and therefore needs to be measured as consistently and accurately as possible. This is even more important when comparing monospecific and mixed stands.

*****Aims***:**

The aim of the study is to find combinations of parameters and allometric relationships that can be used to estimate accurately the leaf area of individual trees.

*****Methods***:**

Allometries of the measured leaf area of 194 trees in 12 stands were analysed in order to find variables affecting leaf area. Existing functions from the literature were validated. Finally, models were fitted to find the most appropriate method for estimating leaf area of mixed and monospecific stands of Norway spruce and European larch.

*****Results***:**

Allometric relationships of leaf area to other measurable characteristics of trees vary in different stand types. Besides individual tree dimensions such as diameter and crown surface area, leaf area of Norway spruce is related to stand density, whereas the leaf area of European larch is dependent on the admixture of Norway spruce in the stand.

*****Conclusion***:**

In contrast to models for estimating individual tree leaf area of Norway spruce, models for leaf area of European larch have to consider mixture proportions in order to correctly interpret the growth efficiency of mixed stands.

## Introduction

The crown of a tree is where the tree interacts with the atmosphere, forming one of the key interfaces within the soil-plant-air-continuum (SPAC). Since leaves are organs in which fundamental physiological processes take place, it is often essential to know the leaf area when studying the growth of individual trees. Light availability is one of the central factors that drive or limit tree growth; Gspaltl et al. (2013) found that light use efficiency could be sufficiently approximated by leaf area efficiency. This means that leaf area is a valuable measure for the evaluation of growth efficiency (see Waring et al. (1980), Binkley and Reid (1984), O’Hara (1988, 1996), Berrill and O’Hara (2007) and Gspaltl et al. (2012)).

To estimate leaf area in the context of studying growth efficiency, it is necessary to use non-destructive methods in order to avoid the measurement method itself affecting growth; one such problematic method would be measuring sapwood area, which is usually done by taking increment cores. Instead, we have to rely on parameters that can be measured without consuming living tissues of the tree. Suitable parameters include basic tree dimensions such as diameter or height, and also measurements of the crown, e.g. crown projection area or crown surface area (for further covariates see Jones et al. (2015)). In some cases, stand characteristics such as dominant height have been found to be significant (cf. Laubhann et al. 2010; Gspaltl and Sterba 2011). Stand variables are generally easier to determine from monospecific stands than mixed stands. For example, the dominant height according to Assmann (1961) is the mean height of the 100 largest trees by diameter at breast height (dbh), irrespective of tree species. If one species of the mixed stand does not occur within the 100 largest trees, the dominant height cannot be determined for this species. Thus, integrating stand characteristics into the leaf area estimation method in mixed stands requires more sophisticated models.

For Norway spruce (*Picea abies* L. Karst.), there are numerous models describing leaf biomass for monocultures (e.g. Jokela et al. 1986; Gower et al. 1993; Lehtonen et al. 2004; Wirth et al. 2004; Eckmüllner 2006; Hochbichler et al. 2006; Ho 2010; Gspaltl and Sterba 2011), but it is not clear whether these models would work as well for mixed stands. For European larch (*Larix decidua* Mill.), there are only a few investigations for leaf biomass in monospecific stands (e.g. Burger 1945; Gower et al. 1993; Rubatscher et al. 2006) with hardly any analysis of mixed stands. If the goal is to compare growth efficiency of European larch and Norway spruce in monospecific and mixed stands, we need leaf area estimation methods that are accurate across different mixing proportions, to ensure that apparent differences in growth efficiency between mixed and monospecific stands do not simply reflect bias in the leaf area estimates rather than real mixing effects on growth.

With the aim of developing such a robust model for estimation of leaf area, we investigated the leaf area of European larch and Norway spruce in stands with different proportions of the two species in order to identify allometric relationships that reflect the quantitative effect of mixture on the leaf area estimation. In particular, we intended to

i) Identify existing models which are able to describe these potential differences, and
ii) Test additional tree and stand variables to see if they improved the performance of the estimates, particularly when applying the same model to stands with different mixture proportions.

Our starting hypothesis was that the effects of mixture on leaf area should be sufficiently described by crown measures, especially by crown surface area. If this presumption is correct, no additional stand variable, e.g. mixing proportion or stand density, would be needed to estimate leaf area and consequently its inclusion in the estimation models would not significantly improve the leaf area estimates.

## Material and methods

### Study area and selected stands

The study area was located in Styria (Austria), near the city of Leoben, approximately at 47° 26′ east latitude and 15° 05′ north longitude. The usual management of stands in this region are strip clearcuts. After clear felling, the forest naturally regenerates in three strips: in the outer strip, European larch prevails while in the inner strip, Norway spruce predominates and in a transitional zone, mixed stands of both species develop.

For our study, we defined four sets of three plots each. Each triplet consisted of one plot in the interior strip, one in the outer strip and one at the edge. In total, 3 stand types (triplets) × 4 replicates = 12 plots were established, so that a total of 4019 trees were measured. The plots in the interior and outer strips were intended to be monospecific; however, it was very difficult to find pure monospecific stands and the plots finally selected contained a range of spruce proportions from 0.02 in the interior strips to 0.93 in the outer strips (Table 1). For simplicity, we will refer to these plots as “monospecific”, though they are not 100% pure.

**Table 1:** Mean plot characteristics

Triplet	Plot	Mixture	Plot size (ha)	Age (years)		MAI_100_ (VfmD/J)		*h*_dom_ (m)		dg (cm)		Prop_Spruce_	Stock_RDI_
				Sp	La	Sp	La	Sp	La	Sp	La		
1	1	Spruce	0.2504	40	–	10.1	–	18.7	–	21.2	–	0.929	0.812
	2	Sp La	0.2664	42	45	9.7	8.9	19.0	21.5	15.2	27.3	0.400	0.743
	3	Larch	0.1379	–	47	–	8.5	–	21.2	–	22.8	0.392	1.010
2	4	Spruce	1.6172	100	–	15.3	–	39.7	–	47.2	–	0.867	0.694
	5	Sp La	1.1660	126	93	9.0	10.0	33.9	34.8	35.2	36.4	0.408	0.634
	6	Larch	0.6805	–	92	–	8.9	–	31.7	–	30.7	0.207	0.750
3	7	Spruce	0.6097	124	–	8.2	–	32.0	–	38.5	–	0.612	0.868
	8	Sp La	0.3281	123	134	8.5	7.8	32.6	33.5	40.5	44.5	0.464	0.977
	9	Larch	1.1543	–	150	–	9.6	–	39.3	–	46.0	0.192	0.758
4	10	Spruce	0.7086	116	–	5.9	–	27.1	–	34.4	–	0.720	0.682
	11	Sp La	1.2886	175	183	6.1	6.7	29.3	31.9	39.7	48.3	0.391	0.714
	12	Larch	1.1104	–	147	–	8.7	–	32.1	–	35.6	0.017	0.680

Mean annual increment at age 100 (MAI_100_), dominant height (*h*_dom_) according to Assmann (1961) and quadratic mean diameter (dg). Proportion of spruce (Prop_Spruce_) and stocking degree (Stock_RDI_) were calculated following Dirnberger et al. (2016) by applying relative density index using potential densities according to Vospernik and Sterba (2014). Mixed stands of Norway spruce and European larch are abbreviated by Sp La, where the values for Norway spruce (Sp) are left lines and for European larch (La) at the right side, respectively

The three plots of each triplet had an approximately similar age and were at the same altitude (details in Table 1); all of the triplets were located between 900 and 1300 m above sea level. The plots were located on slopes between 50 and 70% and exposed from North to Northwest (see Table 1). The average age of trees in each plot ranged from 40 to 183 years. The annual mean temperature and precipitation were 5.2 °C and 1510 mm, respectively (ZAMG 2016; observation period from 2001 to 2013). The soils were determined to be podzolic brown soils (Kilian et al. 1994). Mean values of several measurements are listed in Table 1, showing that the site factors do not vary considerably within the triplets.

The proportion of spruce and stocking degree in Table 1 were calculated with the relative density index as described by Dirnberger et al. (2016). For the calculation, the ratio of the observed stem number (all trees of a plot) to the maximum stem number has to be obtained for each species. The sum of these ratios over all species is the stocking degree and the share of this total for a species gives its mixing proportion (i.e. the ratio of observed to maximum stem number for Norway spruce divided by the stocking degree results in the proportion of spruce). Adequate maximum stem numbers for the species were derived from the maximum density lines of Vospernik and Sterba (2014), based on the data of the Austrian National Forest Inventory.

### Determination of conventional tree and crown measures

The diameter at breast height (dbh), the total tree height as well as the height to crown base were measured at each tree. To determine crown width, we used plumbings of 6–8 points of the crown border, depending on crown shape, using a laser measuring equipment. Crown projection area (i.e. the horizontal projection of the crown) was derived from the crown width.

In order to get a three-dimensional measure of the crown, we estimated crown surface area using species-specific crown shape models (Pretzsch 2009, p. 235).

### Sample tree selection

We felled 15 European larch trees and 10 Norway spruce trees within each mixed stand and the same number of each species in its respective monospecific stand. To select trees for felling, we first measured the diameters (dbh) of all trees in a plot and sorted each species into three dbh classes of equal stem numbers. We then randomly selected trees without damage or breakage within each class. We felled five trees per class for European larch. For Norway spruce, we felled three trees from the upper and lower classes and four trees from the middle class. In total, we sampled 120 European larch and 74 Norway spruce trees.

### Leaf area determination of sample trees

We collected sample branches from the standing trees before felling (to avoid damage to them) and used these branches to analyse leaf allometries. Each sample tree crown was divided vertically into thirds. Within each third, a sample of eight branches was randomly selected and cut exactly 2 cm from the base of the branch. At this point, the branch base diameter was measured, not only for the sample branches but also for every branch of the tree. While the fresh mass was weighed for all sample branches, a sub-sample of four randomly selected branches were cut up to separate the woody parts and the twigs bearing needles (the green mass). For one of these branches, another sub-sample of approximately 200 g was taken to the laboratory to determine dry mass. The twigs were dried for 1 day at 50 °C, so that the needles could easily be separated from the remaining woody parts. The pure needles were dried at 105 °C to constant mass. Following Laubhann et al. (2010), we calculated the needle mass of each branch of the whole tree using allometric relationships. Fresh mass of each branch was determined by using the individual tree relationship to branch base diameter, and the green part of the branch was calculated as a function of fresh mass. We determined the share of dry needles for each branch using the laboratory samples. This procedure was applied to each crown third separately, resulting in precise estimates of dry needle mass. The coefficients of determination (*R* ^2^) of the separate steps ranged from 0.84 to 0.93.

To determine the specific leaf area (SLA), another sample of each crown third was taken containing exactly 50 needles. These needles were scanned and analysed for their projected area. Afterwards, they were dried to constant mass at 105 °C. This procedure, which is described in detail for the European larch samples by Fellner et al. (2016), was applied analogously to all Norway spruce samples. In that study, the relationship between SLA, branch height and crown depth was significant, but resulted only in an *R* ^2^ of 0.22. Therefore, we calculated the projected leaf area of the whole sample tree by applying the SLA of the respective crown thirds.

### Validation of published leaf area models

For Norway spruce, a considerable number of descriptions of biomass functions were available in the literature. Most functions used only diameter to predict the leaf area of the tree. Following Thurnher et al. (2013), we avoided models based on diameter alone because of their limited reliability. We chose the model of Laubhann et al. (2010) that used a three-dimensional measure of the crown (crown surface area) and a stand variable (dominant height) in addition to the diameter at breast height of the tree (dbh). For European larch, we found only a few biomass models in the literature. The model we chose was that of Rubatscher et al. (2006) that predicts the dry needle mass of European larch using dbh and crown ratio.

Both functions we selected use crown allometries to predict leaf biomass and consequently were thought to be appropriate for comparisons with the allometries detected in our stands.

### Statistical analyses

In order to test the performance of the fitted models, we compared the measured and the predicted leaf areas in two steps. First, we calculated *R* ^2^ to test the goodness of fit. Then, we performed simultaneous *F* tests to test whether there was a linear relationship between measured and predicted values; for these tests, we used the null hypothesis that this relationship had an intercept of 0 and a slope of 1.

To develop our own model for estimating leaf area, we followed the suggestions of Laubhann et al. (2010), who introduced the use of crown surface area for predicting leaf area of Norway spruce. Therefore, our first model is described by the following equation

1 $$ \ln {LA}_{\mathrm{Spruce}}\kern0.5em =\kern0.5em  a\kern0.5em +\kern0.5em  b\kern0.5em \cdot \kern0.5em  \ln CSA $$

where *LA*_Spruce_ is the leaf area, *CSA* is the crown surface area and *a* and *b* are coefficients estimated by a log-linear regression. Subsequently, the model selection progressed by adding further tree variables, like dbh and several stand variables. The plots were considered as a random effect in a mixed effects model. In order to have one general equation, we used the function described in Eq. 1 for Norway spruce as well as for European larch. Logarithmic data transformation was applied in order to avoid heteroscedasticity.

We then experimented with adding a series of different stand variables (e.g. proportion of spruce, proportion of larch, dominant height or stocking degree) to test whether these improved the performance of the estimates, and also tested the alternative tree variables crown projection area and crown length. All statistical analyses were performed with the statistical software R (R Core Team 2015). The significance of each new variable or new interaction was tested using the function “anova.lme” to perform Wald tests in the R package “nlme” (Pinheiro et al. 2015). A significance level of α = 0.05 was set as a threshold to keep each variable. When a variable was no longer significant, it was omitted from the subsequent model selection.

The best model was determined by considering several parameters: Akaike’s information criterion (AIC), pseudo-*R* ^2^ and variance inflation factor. The mixed effects models were fitted using the package “nlme” (Pinheiro et al. 2015).

## Results

### Allometric relationship between leaf area and dbh for the sample trees

**Fig. 1:**
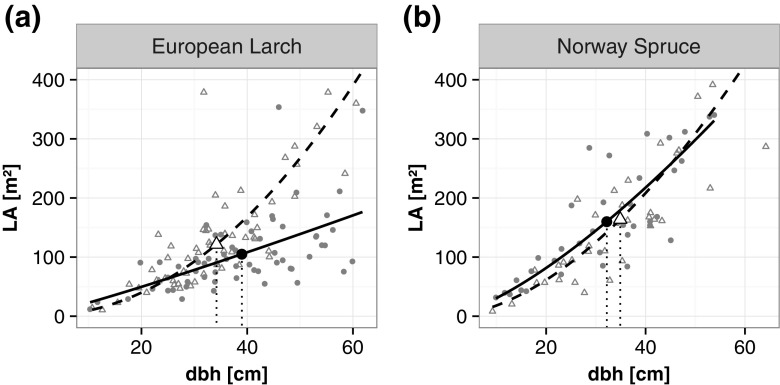
Leaf area (*LA*) as allometric relationship with dbh separated by species. Mixed stands are indicated by *circles* and *solid lines*, monospecific stands by *triangles* and *dashed lines*. The *bold circles* and *triangles* represent the means of the species

Figure 1 shows allometries for leaf area depending on dbh separated by tree species. For European larch, differences between trees in mixed stands (solid lines) and monospecific stands (dashed lines) were immediately apparent. The differences were highly significant (*p* ≪ 0.001), meaning that the estimation of leaf area efficiency based on a common model would lead to over- or under-estimation. For Norway spruce, in contrast, the differences were small and not significant (*p* = 0.608). This indicates that a model for estimating leaf area of European larch would need one or more additional parameters characterizing the mixture.

### Using existing models for estimating leaf biomass

**Fig. 2:**
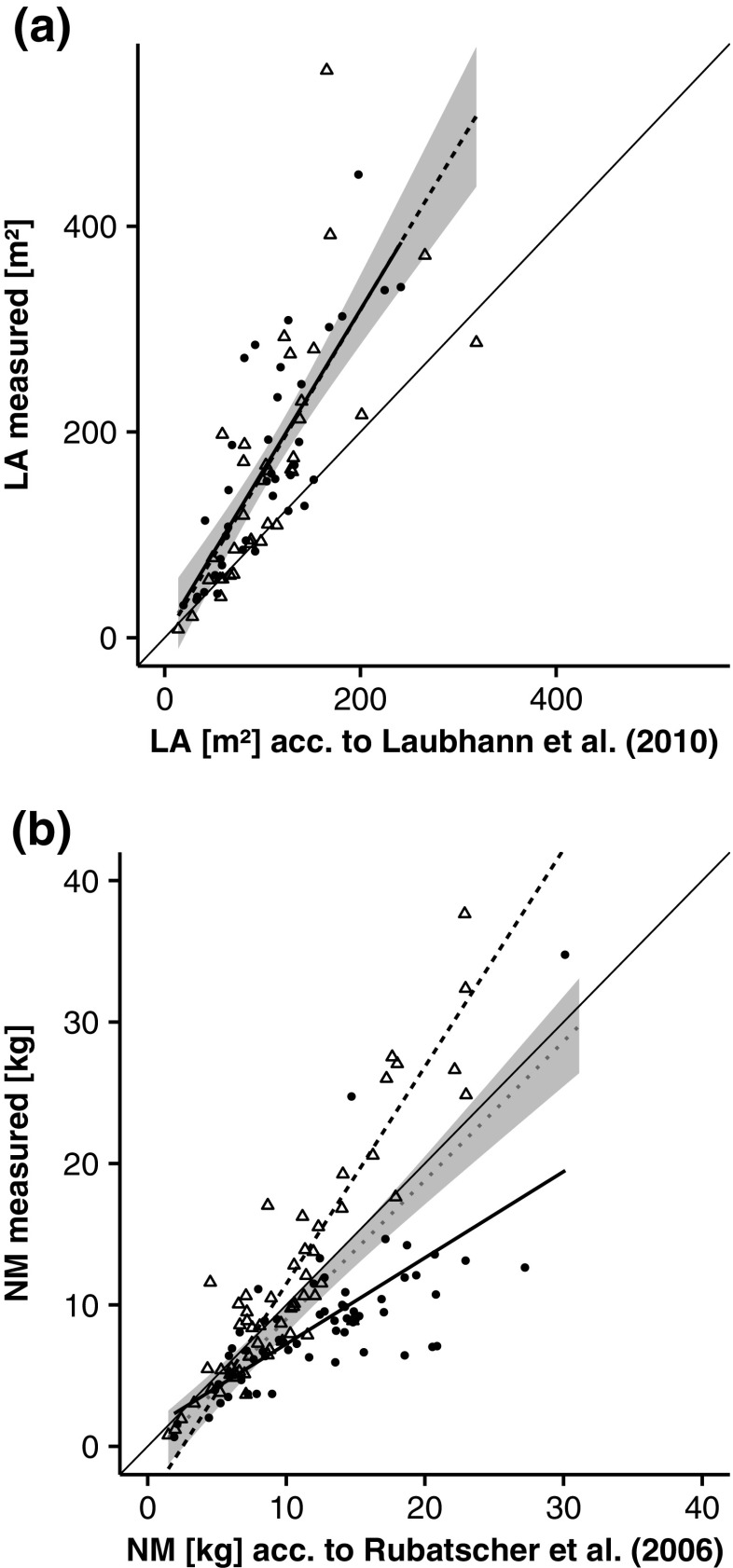
Comparison of measured and predicted leaf area (*LA*) for Norway spruce (**a**) according to Laubhann et al. (2010) and dry needle mass (*NM*) for European larch (**b**) according to Rubatscher et al. (2006). *Circles* and *solid line* represent the mixed stand, *triangles* and *dashed line* the monospecific stand and the *dotted line* represents the overall regression with confidence interval (*grey*)

Our starting hypothesis was that conventional crown measures could sufficiently represent the influence on leaf area, independently of the stand and site characteristics. Therefore, we used the model of Laubhann et al. (2010) for leaf area of Norway spruce and the model of Rubatscher et al. (2006) for needle mass of European larch to see if they complied with our observations (see Table 2 and Fig. 2). These two models already contained crown surface area and crown ratio, respectively, as predictors.

**Table 2:** Inference-statistics on validation of the models for leaf area (LA) of Norway spruce and needle mass of European larch

Species	Model	*R* ^2^	*F*_simult_	*p* (>*F*)
Norway spruce	Laubhann et al. (2010)	0.761	27.78	<<0.001***
European larch	Rubatscher et al. (2006)	0.615	0.777	0.464 ^n.s.^

The *p* values indicate highly significant (***) and no significance (*n*.*s*.), respectively

Although Laubhann et al.’s (2010) model yielded a good correlation with the measured values, it clearly underestimated the leaf area of Norway spruce (Fig. 2a) and resulted in highly significant deviations between the estimated and the measured leaf area (Table 2). The regression lines for mixed and monospecific stands did not differ from the regression line for all stands together. This would be in line with the allometric relationship for Norway spruce trees in our study.

Rubatscher et al. (2006) did not estimate leaf area of European larch directly; rather, they measured dry needle mass. Their model led to good estimates when applied to the overall population of trees in our stands. The overall regression model showed non-significant deviations when a simultaneous *F* test (Table 2) was applied. However, this model underestimated the needle mass in monospecific stands and overestimated it in mixed spruce-larch stands (Fig. 2b). These results show that to correctly estimate leaf area of European larch in stands with different mixture proportions, it is necessary to use an additional predictor for stand type.

### Developing a site-specific model for estimating leaf area

Based on the preliminary results (Figs. 1 and 2), we followed Laubhann et al. (2010) and fitted a model for leaf area depending on crown surface area and dbh, with additional variables characterizing stand type. The model selection procedure resulted in several plausible models, from which we ultimately chose models in which all coefficients were significant (*α* = 0.05). Within these best models, crown surface area and dbh were included for both species. Additionally, the proportion of Norway spruce turned out to have a significant effect on the leaf area of European larch, whereas the leaf area of Norway spruce was affected by density as indicated by stocking degree.

The fitted linear mixed effects models for Norway spruce and European larch are shown in Eqs. 2 and 3.

2 $$ {LA}_{\mathrm{Spruce}}\kern0.5em =\kern0.5em { \exp}^{\beta_0\kern0.5em +\kern0.5em {\beta}_1\kern0.5em \cdot \kern0.5em  \ln CSA\kern0.5em +\kern0.5em {\beta}_2\kern0.5em \cdot \kern0.5em  \ln dbh\kern0.5em +\kern0.5em {\beta}_3\kern0.5em \cdot \kern0.5em {Stock}_{RDI}\kern0.5em +\kern0.5em {u}_{\mathrm{Spruce}}}\kern0.5em \cdot \kern0.5em {\lambda}_{\mathrm{Spruce}} $$

3 $$ {LA}_{\mathrm{Larch}}\kern0.5em =\kern0.5em { \exp}^{\beta_0\kern0.5em +\kern0.5em {\beta}_1\kern0.5em \cdot \kern0.5em  \ln CSA\kern0.5em +\kern0.5em  \ln dbh\kern0.5em \cdot \kern0.5em \left({\beta}_2\kern0.5em +\kern0.5em {\beta}_3\kern0.5em \cdot \kern0.5em \mathit{\Pr}{op}_{Spruce}\right)\kern0.5em +\kern0.5em {u}_{Larch}}\cdot {\lambda}_{\mathrm{Larch}} $$

For estimating leaf area (LA), we found that the fixed effects crown surface area (CSA) in m^2^, diameter at breast height (dbh) in cm and stocking degree (Stock_RDI_) or proportion of Norway spruce (Prop_Spruce_) are needed. Finally, *u* is the random effect of the plots and *λ* is the correction factor, necessary due to the logarithmic data transformation. For the statistics of these final equations, see Table 3.

**Table 3:** Estimated coefficients of the linear mixed effects model for each tree species (see Eq. 2 for Norway spruce and Eq. 3 for European larch, respectively) with *β*_*i*_ the coefficient of the *i*th fixed effect and the respective *p* values (in parenthesis)

Species	*β*_*0*_	*β*_*1*_	*β*_*2*_	*β*_*3*_	*λ*	*R* ^2^ *m*.	*R* ^2^ *c.*	*σ*_*u*_^2^	*σ*_*ε*_^2^
Spruce	−0.3385 (0.5608)	0.5577 (0.0014)	1.0267 (<<0.0001)	−1.3102 (0.0384)	1.0341	0.8439	0.8613	±0.1062	±0.2994
Larch	−0.9046 (0.0360)	0.3867 (0.0072)	1.0841 (<<0.0001)	−0.3207 (0.0256)	1.0597	0.6921	0.7610	±0.1881	±0.3503

*λ* is the correction factor due to the logarithmic transformation. *R* ^*2*^ *m.* represents the marginal coefficient of determination and *R* ^*2*^ *c.* the conditional one. σ_u_^2^ is the variance of the random effect and σ_ε_^2^ is the residual variance

The stand variables affecting leaf area differed for the two species. While leaf area of Norway spruce was significantly affected by stand density in terms of stocking degree, for European larch, the admixture of Norway spruce had a significant effect, rather than density. To exclude confounding between the effect of density and mixing proportion, we tested their correlation, which turned out to be near zero (*R* ^2^ = 0.0038, see Table 1).

**Fig. 3:**
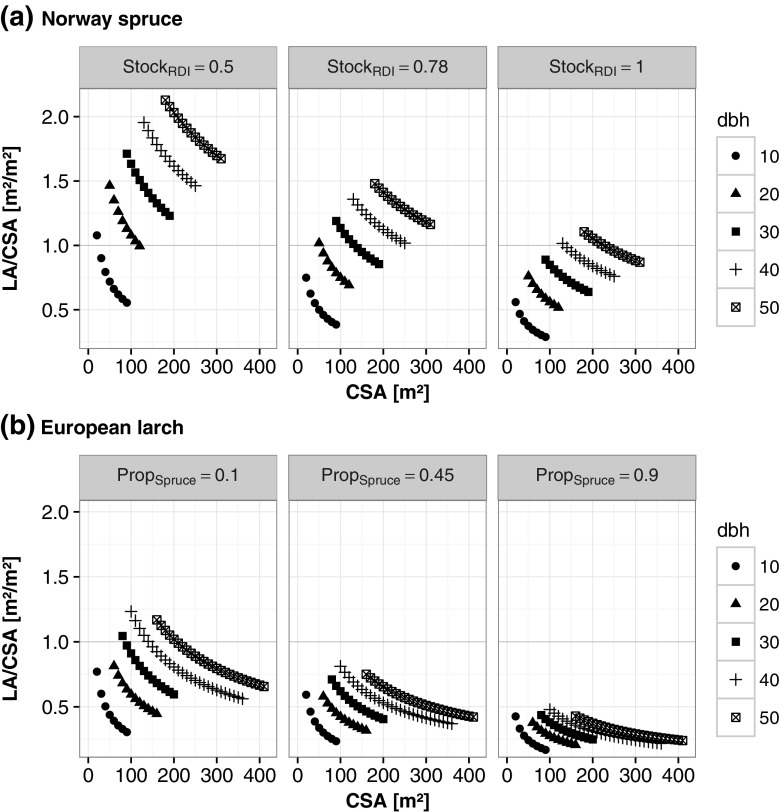
Leaf area per crown surface area (*LA*/*CSA*) dependent on crown surface area (*CSA*) grouped by dbh, derived from the spruce model (**a**) for low, average and high stand density (Stock_RDI_), and derived from the larch model (**b**) for low, average and high proportion of spruce (Prop_Spruce_) within our data range (thereby average represents the mean value of all stands)

We observed the following trends for leaf area of Norway spruce from Fig. 3a:

- Leaf area per crown surface area (crown density) increased with decreasing stocking degree.
- At a given stocking degree, crown density increased strongly with the dbh.
- At a given dbh, crown density decreased with increasing crown surface area.

Similarly, we observed the following trends for European larch from Fig. 3b:

- Crown surface area and dbh had a similar effect as for Norway spruce.
- Increasing proportions of Norway spruce in the stand resulted in a decrease of leaf area per crown surface area (i.e. a less-dense crown). On closer examination, this effect is more complex: crown density increased with increasing dbh, but the slope of this increase decreased with higher proportions of Norway spruce.

An average stand in our data range would have a stocking degree of 0.78 and a spruce proportion of 0.45 (see middle panel of Fig. 3). In this average stand, Norway spruce trees would have more leaf area per crown surface area than the European larch trees. This result was not unexpected due to the fact that Norway spruce does not shed its needles for several years. However, the slope of increase over all dbh-classes is much steeper for Norway spruce than for European larch.

## Discussion

Numerous studies have been conducted to compare trees in mixed versus monospecific stands. Most of these investigations focused on comparisons of productivity (e.g. Kelty 1992; DeBell et al. 1997; Chen et al. 2003; Bristow et al. 2006; Río and Sterba 2009; Bielak et al. 2014; Condés and Río 2015; Pretzsch et al. 2015). Measured growth of a tree (e.g. in terms of volume or biomass increment) is often related to one or another measure of the available growing space (e.g. leaf area, O’Hara 1988; Gspaltl et al. 2012). Considering that most studies do not focus on the determination of leaf area, there is an inherent problem. If the estimates of leaf area are inaccurate, the leaf area efficiency, and consequently the comparisons of mixed and monospecific stands, may become biased. Forrester and Pretzsch (2015) already discussed the potential for bias resulting from incorrect allometric relationships.

We found considerable differences when investigating leaf area allometries with individual tree measurements and stand characteristics. While Wirth et al. (2004) and Genet et al. (2011) already emphasized the importance of stand characteristics for leaf area estimates, they considered only additional tree variables like dbh or height in their models. Interestingly, our results show that even after we include the crown measures, with the aim of representing all the variations in crown architecture, stand variables are still significant. However, the two tree species also had different relationships. While the allometries for leaf area of Norway spruce did not differ significantly between mixed and monospecific stands, for European larch, the allometries for leaf area resulted in significant differences for the different stand types (Fig. 1). Therefore, evaluating leaf area efficiency in mixed stands using leaf area equations derived from monospecific stands is likely to generate inaccurate results.

Figure 1 also suggests that European larch trees have more leaf area in monospecific than in mixed stands and thus have a higher potential for productivity and growth. This is actually true for a given dbh and crown surface area. However, a simple inference that the trees are more productive in monospecific stands is partly contradicted by the finding that the mean diameters of European larch trees are higher in the mixed stands than in the monospecific stands (mean values in Fig. 1). In any case, models for estimating leaf area need to reflect these differences in crown allometries. The model of Rubatscher et al. (2006) for European larch does not account for these factors. Although it was able to provide plausible values when applied as an overall model for all stand types, it failed when the data were split into mixed and monospecific stands (Fig. 2b). In contrast, the model of Laubhann et al. (2010) for Norway spruce led to overall underestimates of the measured values (Fig. 2a) as well as for the different stand types.

To overcome these limitations, we fitted site-specific models for predicting leaf area. In contrast to other studies (e.g. Shinozaki et al. 1964; Waring et al. 1982; Eckmüllner and Sterba 2000), we did not use sapwood area to estimate leaf area, for two reasons. Firstly, the tested models including sapwood area performed worse than the ones using crown surface area (in terms of pseudo-*R* ^2^ and residual variance). Secondly, the repeated invasive investigations of trees, including coring for sapwood area, may distort future observations because it leads to the formation of a wound meristem. Methods for estimating leaf area need to be strictly non-destructive if the measurements are being associated with the growth of the tree. Therefore, we used crown surface area as the main predictor variable. Laubhann et al. (2010) already found that this variable was the best predictor for estimating leaf area of Norway spruce. We estimated the crown surface area using crown shape models according to Pretzsch (2009). Since diameter was not included in these models, we expected to improve the leaf area models by including dbh, since this measure was supposed to account for the developmental stage of the tree.

Several further variables were tested to see whether they also could improve the models. For each species, just one other variable was found to contribute to the quality of the estimates: for European larch, this variable was the proportion of Norway spruce in the stand and for Norway spruce it was stocking degree.

Contrary to our starting hypothesis, the estimation of the leaf area of European larch is affected significantly by the mixing proportions as well as by crown dimensions. Interestingly, the stocking degree was significant for Norway spruce but not for European larch. The reason for this result may be found in the completely different traits of these species with respect to light. The higher demand for light by European larch causes them to be somewhat higher than Norway spruce trees (at least in terms of dominant height). Therefore, the crown and particularly the leaf area of European larch might not be affected by density—at least not to an extent that was detectable in our data range.

## Conclusion

While leaf area for Norway spruce can be estimated without taking account of mixing proportions in the stands, the same is not true for European larch. The leaf area of individual European larch trees with otherwise equal dimensions is higher in the absence of Norway spruce than in mixed stands.

Functions found in the literature for estimating leaf area of Norway spruce do not seem to be applicable to the sites of our investigation. The model for predicting leaf mass of European larch by the equations of Rubatscher et al. (2006) provides plausible values on average over the different stand type, but on closer analysis, this model turned out to overestimate the leaf biomass in mixed stands and underestimate it in monospecific stands.

An adequate model for estimating leaf area should contain crown surface area and dbh. For European larch, an additional measure for stand type is needed (proportion of Norway spruce) to account for the mixture-related differences. For Norway spruce, on the other hand, the mixing proportions were irrelevant but a measure of density (stocking degree) made a significant additional contribution to the estimates. Thus, only the leaf area of European larch is affected by mixture. Although we did not have 100% pure monospecific stands in the samples, the range of spruce mixing proportions was wide enough to conclude that our models will be useful for interpreting growth efficiency in monospecific versus mixed stands of European larch and Norway spruce.
